# The impact of remote care approaches on continuity in primary care: a mixed-studies systematic review

**DOI:** 10.3399/BJGP.2022.0398

**Published:** 2023-03

**Authors:** Emma Ladds, Maaedah Khan, Lucy Moore, Asli Kalin, Trish Greenhalgh

**Affiliations:** Nuffield Department of Primary Care Health Sciences, University of Oxford, Oxford.; Medical Sciences Division, University of Oxford, Oxford.; Nuffield Department of Primary Care Health Sciences, University of Oxford, Oxford.; Nuffield Department of Primary Care Health Sciences, University of Oxford, Oxford.; Nuffield Department of Primary Care Health Sciences, University of Oxford, Oxford.

**Keywords:** consultation, continuity of care, primary care, remote consultation

## Abstract

**Background:**

The value of continuity in primary care has been demonstrated for multiple positive outcomes. However, little is known about how the expansion of remote and digital care models in primary care have impacted continuity.

**Aim:**

To explore the impact of the expansion of remote and digital care models on continuity in primary care.

**Design and setting:**

A systematic review of continuity in primary care.

**Method:**

A keyword search of Embase, MEDLINE, and CINAHL databases was used along with snowball sampling to identify relevant English-language qualitative and quantitative studies from any country between 2000 and 2022, which explored remote or digital approaches in primary care and continuity. Relevant data were extracted, analysed using GRADE-CERQual, and narratively synthesised.

**Results:**

Fifteen studies were included in the review. The specific impact of remote approaches on continuity was rarely overtly addressed. Some patients expressed a preference for relational continuity depending on circumstance, problem, and context; others prioritised access. Clinicians valued continuity, with some viewing remote consultations more suitable where there was high episodic or relational continuity. With lower continuity, patients and clinicians considered remote consultations harder, higher risk, and poorer quality. Some evidence suggested that remote approaches and/or their implementation risked worsening inequalities and causing harm by reducing continuity where it was valuable. However, if deployed strategically and flexibly, remote approaches could improve continuity.

**Conclusion:**

While the value of continuity in primary care has previously been well demonstrated, the dearth of evidence around continuity in a remote and digital context is troubling. Further research is, therefore, needed to explore the links between the shift to remote care, continuity and equity, using real-world evaluation frameworks to ascertain when and for whom continuity adds most value, and how this can be enabled or maintained.

## INTRODUCTION

Primary care, the *‘provision of integrated, accessible health care services by clinicians who are accountable for addressing a large majority of personal health care needs, developing a sustained partnership with patients, and practicing in the context of family and community’,*^1^^,^^2^ has four key attributes. These are first-contact access; a long-term person focus (continuity); comprehensiveness; and coordination; with a secondary focus on family-centredness, cultural competency, and community orientation.^3^^,^^4^ Healthcare systems with strong primary care sectors are associated with improved and more equitable health outcomes and greater cost efficiencies than those more reliant on specialist services.^5^^,^^6^

Continuity of care is an overarching concept that conveys a tacit sense of stability, consistency, and connection in the relationships and processes that comprise the experience of health care. Over the years, continuity has been understood in a range of ways, from ‘seeing the doctor you know and trust’ to ‘the experience of a coordinated and smooth progression of care’.^7^ Changing definitions have added to the challenge of investigating, analysing, and communicating the value of continuity, contributing to it receiving a ‘softer’ status than, for example, waiting times or quality metrics.^8^

Broadly, four overlapping elements of continuity have been delineated: interpersonal or relational continuity between clinicians and patients; longitudinal continuity, occurring over time, potentially between providers and sometimes limited to particular care episodes (episodic continuity); management continuity, which may involve collaboration or proactive planning; and informational continuity, using records to communicate a shared understanding of an individual.^9^ Higher levels of continuity are associated with numerous benefits including improved coordination of care and fewer unnecessary secondary care episodes,^10^^,^^11^ better outcomes such as medication adherence and diabetes control,^12^ reduced mortality rates,^13^ and patient and doctor satisfaction.^9^^,^^14^^,^^15^

For generations, continuity has formed a tacitly understood cornerstone in the relationship between GPs and patients that rarely required an overt acknowledgement.^7^^,^^16^ However, fragmentation and destabilisation of societies and communities, combined with an increased policy emphasis on rapid access, plurality of provision, and ever-greater specialisation, has resulted in a reduced emphasis on continuity in UK general practice,^8^^,^^16^ with declining levels among practices,^17^ and claims that it is *‘going out of style’*.^18^ Recently, there has been greater recognition of the costs of declining continuity rates;^19^ however, it is likely to be a challenging tide to turn.

**Table 1:** How this fits in

The value of continuity in primary care has been repeatedly demonstrated for multiple positive outcomes. However, little is known about how the expansion of remote and digital care models has impacted continuity. This study demonstrates a disturbing lack of systematic research in this area and emphasises the need for real-world explorations of the links between the shift to remote care, continuity, and equity to ascertain when and for whom continuity adds most value, and how this can be enabled or maintained.

In recent years, the complexities surrounding continuity have been exacerbated by the transformative move to digital-first approaches. Before the COVID-19 pandemic, remote care (telephone or video consultations, or asynchronous digital care encounters)^20^^,^^21^ was still in its infancy,^22^ although early work looking at telephone consultations, email contacts, and text messages had concluded that they could aid continuity if deployed appropriately, and in the context of pre-existing relationships.^23^ Larger studies were underway to explore alternatives to face-to-face consultations,^24^ with some opinion pieces raising concerns about their impact on continuity and therapeutic relationships.^22^

However, in 2020, the COVID-19 pandemic led to widespread deployment of remote care approaches,^25^ which have persisted.^26^^,^^27^ Combined with system changes such as larger-scale collaborative working and a more multidisciplinary workforce,^28^ these are likely to have had significant impacts on continuity. Indeed, a 2021 systematic review highlighted concerns about the potential of widespread remote consulting to worsen inequities in general practice and the limited exploration to date of the quality of care and outcomes of such approaches.^29^

While it is possible to discretely dissect components of continuity, the complexity of interacting, contextually specific influences, and the downstream impacts relating to its presence (or absence) are harder to unpick. The experience of continuity of care for patients, professionals, and wider society has an emergent value and virtue, which is far harder to define and quantify. As Tsoukas pointed out,^30^ by attempting to simplify the discussion around complex subjects we lose their ‘real-world’ meaning or significance, and resultant theoretical inferences are less helpful. This systematic review therefore set out to explore the published evidence around continuity in this new era of remote care and, alongside identifying specific influences or downstream results from changes to the traditional model, to try and consider whether wider complexities are being explored, which may enable such conjunctive theorising about the broader impacts of such alterations.

Remote care in this review is defined as any encounter (synchronous/asynchronous) that does not take place in person between a patient and primary care team, and includes explicit references to any aspect of continuity.

## METHOD

### Management and governance

This study was conducted as part of the ‘Remote by Default 2’ research programme (RbD2), which uses mixed methods to explore the application and complexities of practised remote care in UK general practice across a number of cross-cutting themes. These include, among others, management of long-term conditions, workforce and training, access and inequalities, continuity, infrastructure, and sustainability.

### Search strategy

A keyword search of the electronic databases Embase, MEDLINE, and CINAHL was conducted in January 2022 for English-language studies published after 1 January 2000. The search terms related to ‘primary care’ AND ‘remote consultations’ AND ‘continuity’ (see Supplementary Box S1 for details). Additional citations were sought from reference lists of selected studies and citation tracking. Duplicates were removed and the results exported and managed using EndNote X9 bibliographic software.

### Inclusion and exclusion criteria

Original studies, reviews, quality improvement projects, and case studies were included from any country, provided that they focused on primary care, primary care physicians, or other members of the team directly linked to the main primary care provider, such as diabetes nurses in a general practice. Studies were excluded if they focused on non-community settings, specialist practitioners/services, or those not linked directly to the primary care provider, such as independent community diabetes teams. Studies also had to explore some aspect of remote care (defined as above) and continuity.

### Selection and data extraction

One researcher screened all abstracts for relevance, and then reviewed full articles along with one of the other researchers. Figure 1 shows details of the PRISMA flow diagram. Data were extracted using a template to organise and manage sources, but, given the contextual variation, study immersion and familiarisation with discussion among the research team was also key.

**Figure S1.**
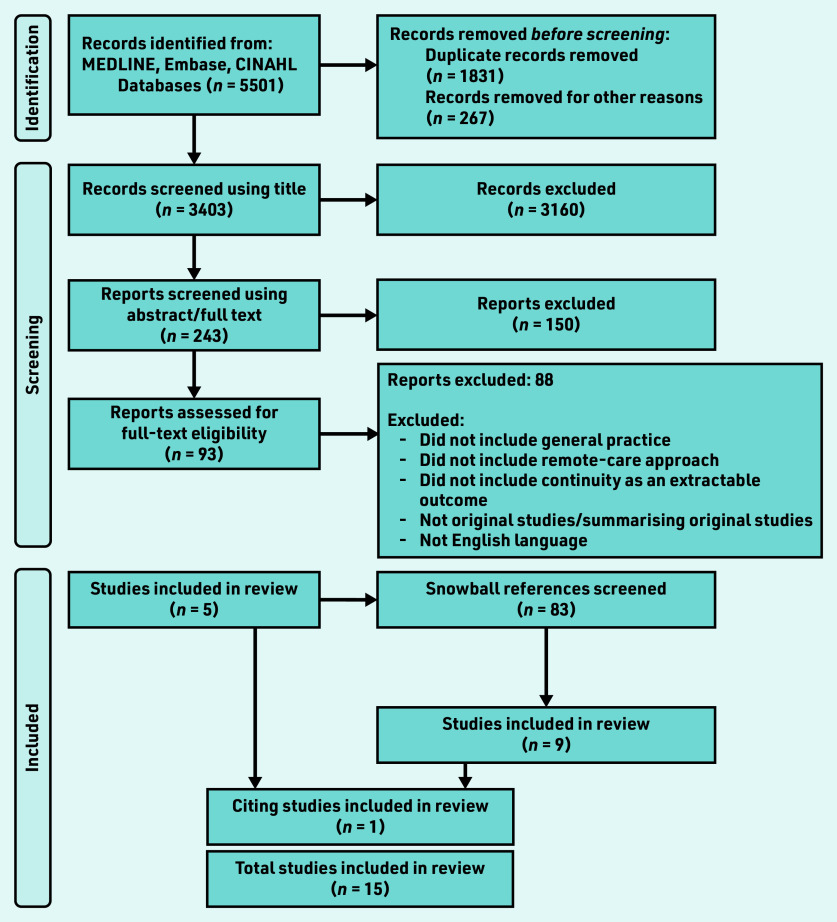
*PRISMA flow diagram.*

### Data management and analysis

EndNote X9 33 software was used to manage and share data. Key themes were highlighted from individual studies with team discussion, constant comparison between studies, and a search for convergent and discordant data (data in agreement or disagreement), which allowed for development of a narrative synthesis. Rigour was strengthened by reflexivity among team members and the confidence of the review findings was subsequently assessed by application of the GRADE-CERQual^31^ and CASP checklists,^32^ which are international standards used to determine confidence in findings from reviews of qualitative studies. CERQual was chosen as the most appropriate assessment standard after the search revealed only one quantitative study for inclusion. Given the inclusivity of the search strategy, the researchers suspect that the lack of quantitative studies might represent a lack of quantitative assessments of continuity, rather than a bias of the search strategy itself.

## RESULTS

### Description of dataset

Of 5501 documents retrieved, 93 were selected for full-text examination, with five contributing data. A further 83 articles were highlighted through snowballing and citation tracking, and nine were selected. The final dataset of 15 articles consisted of four interview studies with patients,^33^^–^36 four with clinicians,^37^^–^40 and one with health policymakers;^41^ two survey studies of clinicians,^42^^,^^43^ and one of patients and clinicians;^44^ two mixed-method studies,^45^^,^^46^ and one quality improvement project report.^47^ Studies were set in a total of eight countries and all were published from 2018 onwards (see Supplementary Table S1 for details).

### Confidence in findings

GRADE-CERQual analysis consists of summarising (Supplementary Table S2) the: quality (Supplementary Table S3), adequacy, and relevance (Supplementary Table S4) of qualitative studies.^48^

### Themes

Many themes highlighted in this review relating to the perceived importance (or not) of continuity, factors influencing its attainment, or downstream consequences are frequently inter-related and not specific to remote approaches *per se*. However, widespread remote approaches may add a level of nuance and complexity to continuity through interactions with wider factors influencing the quality of access and consultations, in particular the underpinning principles of clinical ethics and ethics of care.

#### Sparsity of studies specifically reporting and measuring continuity.

Of the initial 176 studies selected for full-text analysis, it was notable how few looked explicitly at continuity. Most were rejected because it was impossible to differentiate continuity from broader concepts such as ‘pre-existing relationships’ between patients and their primary care teams or a doctor’s ‘prior knowledge’ of their patients. Moreover, few studies differentiated between aspects of continuity. Johnsen *et al*^43^ looked specifically at relational and episodic continuity, while Trabjerg *et al*,^44^ Hansen *et al*,^38^and Tönnies *et al*^41^ reported improved managerial and informational continuity with integrated primary–specialist consultations without referring to it as such, for example, describing *‘more coherent patient trajectory*[ies] *’* and *‘roles and tasks* [becoming] *more apparent to both patients and professionals and* [sharing] *knowledge between health sectors’.*^44^

#### Patient factors influencing continuity of care.

Several studies reported the value some individuals placed on relational continuity with ‘their’ GP or primary care clinician.^33^^–^35 Sometimes this was because of uncertainty with less visible aspects of continuity (such as informational continuity) or because relational continuity itself was valued. For example, one participant with ongoing mental health problems reported their concern that *‘it’s quite complicated and my preferred GP knows me from day one and has worked with me and referred me and supported me … I just didn’t know how much this person knew’.*^33^

In one study, patients who valued relational continuity actively chose a telehealth appointment with their GP over an in-person consultation with a different GP.^36^

#### Health professional factors influencing continuity of care.

Some GPs emphasised the importance of consultations (remote or face-to-face) with known patients,^37^^,^^40^^,^^42^ with some indicating such knowledge was a prerequisite for effective consultations.^42^ Some GPs reported how the flexibility of remote approaches could enhance this continuity with their patients by allowing them to keep their *‘finger on the pulse much more’.*^37^ Johnsen *et al*^43^ used a nationwide survey of Norwegian GPs to quantify the value GPs placed on relational and episodic continuity in determining the suitability of using video consultations. Both measures of continuity resulted in statistically significant higher suitability ratings, suggesting that GPs viewed remote consultations as more suitable for follow-up presentations, particularly in the context of high relational continuity.

#### System factors influencing continuity of care.

Some studies reported improved access to patients’ usual or preferred GP with remote care approaches,^33^^,^^35^ with one patient using an Australian GP telehealth model reporting that *‘in fact, I’m probably seeing him* [the GP] *more now via the phone’.*^35^ However, some also reported a trade-off for patients between continuity and ease or speed of access.^33^^,^^40^^,^^45^ Salisbury *et al*^45^ carried out an independent evaluation of Babylon GP at Hand (BGPaH), a private company offering NHS GP consultations, and found that individuals choosing BGPaH, who were generally young with few long-term health needs, did so because of speed and ease of access, deprioritising continuity. However, some patients with complex needs were concerned about its absence because, as one BGPaH user stated, *‘there’s nothing for long-term health management’.*^45^

Several studies illustrated the strategic development of remote approaches to improve relational, informational, and managerial continuity both within and between the healthcare system. Integrated care consultations, for example, whereby the patient and GP were situated together in the GP practice and conducted a joint remote consultation with an oncologist, were shown to improve understanding of the roles of different specialists (such as cancer and mental health) in the patient’s journey, resulting in a more coherent care pathway and improved managerial continuity.^38^^,^^41^^,^^44^

In two studies, patients, GPs, and oncologists believed that such integrated consultations contributed to better continuity of care and thus health outcomes, with all involved gaining a better understanding of how to optimise managerial and informational continuity,^38^^,^^44^ although such structures may be considered to be pushing the boundaries of traditional general practice activities, relating more to the primary–secondary care interface.

Furthermore, one study reported health policy experts’ opinions that the relationship of trust between a GP and patient, often formed over ‘a long time’ (that is, reflecting relational continuity), could help motivate patients to engage in remote consultations with mental health specialists,^41^ while oncologists reported how the *‘long-established relationships* [between GP and patient] *could help overcome mistrust of specialists or the wider system’ .*^38^

Finally, informational continuity was deployed strategically to contact vulnerable patients proactively by telephone, demonstrating the value of combining continuity and remote approaches.^40^

Other studies reported patients’ concerns about the implementation of remote approaches in systems, such as telephone triage or same-day appointments, which could make it more difficult to access their preferred GP. This resulted in frustration, distress, harm, and increased inefficiencies.^33^^,^^34^^,^^47^

One older male patient with complex conditions reported an attempt to contact his preferred GP: *‘I said what was wrong and that I needed to see the Doctor. She says well Doctor* [X] *is not in today — phone tomorrow. Bump* [phone being hung up]*. So I phoned the next morning at 8 o’clock. Phones off. I phoned every 5 mins till 8.30 am — it came on, “surgery’s now full’, phone Monday … It’s that bad you couldn’t make it up. If they had someone to report it to I’d prosecute them.’*^33^

Similarly, another user of an online platform reported multiple consultations because, *‘I have high blood pressure. I’ve been trying to get in touch with the doctor to explain what I need to do … I’ve had two blood tests in the space of 2–3 weeks and have no idea what’s going on.’*^47^

#### The patient–doctor relationship.

Many patients and healthcare practitioners believed that remote consultations in the context of pre-existing relationships were easier, safer, and of higher quality.^33^^–^35^,^^38^^,^^42^^,^^44^^,^^46^^,^^49^ There was a general recognition that continuity was only one aspect in such relationships, with mutual trust, respect, active listening and communication, compassion, empathy, and rapport building all thought to be important.

Many healthcare professionals considered relational or episodic continuity essential for eliciting the subtleties in patients’ concerns. Verhoeven *et al*^40^ found that, while the focus of a telephone triage may be on obvious complaints, where GPs knew patients well they could detect other aspects. As one GP responder stated, *‘most of the time the consultations are about a physical symptom … but when you ask a bit more you hear they are actually very worried’*. *‘*Very worried’ might represent psychological distress or serious patient or parent concern about any complaint*.* Similarly, Johnsen *et al*^43^ demonstrated that video consultations were deemed more suitable for follow-up consultations rather than first presentations (even when there were high levels of relational and/or episodic continuity), reflecting a concern that remote approaches may miss information that would be obtained in person, which is potentially more important in first presentations.

Remote approaches also affected presentation rates according to whether individuals thought their health needs were met, for example, patients with mental health or chronic conditions reported missing the social cues and body language or struggling with digital systems in times of deterioration. They were concerned about their ability to form a relationship with the clinician, resulting in less satisfying/successful encounters, and reduced presentations.^33^^,^^36^

In contrast, high-frequency users of an online platform — again who often had complex chronic or mental health conditions — perceived a lack of continuity of care, generating repeated consultations because of a perception of unmet health needs.^47^

However, the BGPaH evaluation reported a high level of patient satisfaction, with participants rating that their needs had been met, the clinician had listened and treated them with care and concern, and that they had confidence and trust in the clinician.^47^ Despite methodological concerns about recruitment bias, this suggests that relational continuity is not essential for a successful consultation. This finding was supported in a study by Imlach *et al*,^34^ in which patients reported successful remote consultations in the absence of a pre-existing relationship and unsuccessful ones in its presence, depending on whether an effective rapport was generated.

#### Risks of the impact of remote care on continuity.

Several studies highlighted the potential for remote approaches (or their implementation) to exacerbate inequities of care by reducing relational or episodic continuity for patients who value such care and for whom continuity is likely to significantly impact outcomes, such as those with complex or chronic conditions.^33^^,^^34^^,^^39^^,^^42^^,^^45^

Similarly, Swedish GPs expressed concern at the trade-off between ease of access and a resultant increased workload, which might impair continuity for those needing it by overwhelming the system.^42^ They described digitalisation as a *‘stressful time thief’*, explaining that if *‘health care becomes too accessible only minor ailments can be dealt with. Because health care resources are insufficient, this contact method takes resources from those who need it better’,* in other words, older people with multimorbidity.^42^ Remote approaches targeting long-term conditions, such as asynchronous blood glucose or blood pressure monitoring, could compromise safety if the submission processes did not identify an appropriate clinician with sufficient time to deal with them.^42^

Flexibility was highlighted as essential for effective implementation of remote approaches, with contrasting views as to whether patient choice or need should predominate.^33^^,^^35^^,^^37^^,^^39^

One GP from a practice with a highly deprived population described the problems of universal, centralised, and inflexible policy decisions about technology and access, *‘in terms of the technology that Matt Hancock* [the UK Secretary of State for Health and Social Care at the time] *seems to think is the way forward and just because him and all his peers, you know have access to all the technology, and it’s very convenient for them to consult with their GP via Zoom, that is not how it is for the people where I work’*.*39* Not only did remote care reduce managerial continuity but also generated barriers between the practice and the wider community.

Patients and clinicians also expressed clinical safety concerns with remote approaches, which could be mitigated but not eliminated through continuity. Despite high episodic and relational continuity, and a large number of follow-up appointments, 15% of the video consultations reviewed in Johnsen *et al*’s study^43^ were believed to risk missing serious illness, a sentiment echoed by patients, particularly when they were unable to see their usual GP.^35^^,^^37^ Moreover, continuity cannot make up for technical or contextual factors that limit remote care such as digital poverty, lack of safe spaces for consultations, or the impact of illness itself such as mental health crises. 36^,^^39^

## DISCUSSION

### Summary

In this narrative exploration of remote approaches and continuity in primary care, just 15 of 5584 studies met the inclusion criteria. The most notable finding was the paucity of studies looking explicitly at continuity distinct from the broader concept of a ‘pre-existing’ relationship, although many studies tacitly acknowledged the role that relational or episodic continuity played in facilitating this relationship. Only one study^43^ specifically differentiated between components of continuity and none attempted to measure it. This may be because of the range of possible assessment measures available, or may reflect the perception that continuity is a ‘soft’ measure of general practice quality rather than a potential ‘hard’ metric.

In the context of very limited published research, various factors were identified relating to patients, healthcare professionals, the patient–professional interaction, and the wider system, which appeared to influence (or were perceived by patients or professionals as influencing) the provision of different forms of continuity.

While the themes have been separated for clarity, many are inter-related, for example, system factors may constrain what professionals are able to do to influence continuity of care, which in turn may affect the patient–doctor relationship. Moreover, many of these factors are not unique to remote care approaches. Rather, they overlap and interact with wider influences on the quality of access and consultations, in particular the underpinning principles of clinical ethics and ethics of care.

Ascertaining when continuity really matters, for whom, and how it can best be established should be important considerations in the functional future of general practice. Studies here reveal how some patients, often those with long-term or complex conditions, value relational continuity with their GP, while others will accept a trade-off for convenience or speed of access, at least when their health is stable. Similarly, some professionals particularly value relational and episodic continuity, which could affect their attitude towards remote care — in some cases, the flexibility of remote contacts actively enhanced continuity. System issues, particularly relating to triage and access, were highly significant in affecting patients’ ability to maintain relational continuity when using remote approaches and could result in frustration, distress, and harm for patients, as well as inefficiencies.

A pre-existing relationship and ongoing relational continuity were considered important contributors to (but did not guarantee) a high-quality, safe, and satisfactory remote consultation. Finally, a number of potential risks were identified secondary to impacts on continuity, including worsened health inequities, increased clinical risk, and a detrimental impact on the role of general practice in communities.

### Strengths and limitations

A structured search was used in combination with reference snowballing to identify studies of various designs from eight countries with different primary care systems, which were assessed by multiple researchers, using GRADE-CERQual and CASP, reflection, and team discussions. The narrative approach to synthesis highlighted themes to explore more deeply in the ongoing RbD2 study.^50^ However, no protocol was published in advance and only one reviewer conducted the initial abstract screening. It is also important to note that the snowballing search detected more results than the search itself. This was largely because ‘continuity’ was not specifically referenced in the title or abstract, thus was not detected in the initial search. On closer scrutiny and snowballing, it was possible to identify more articles where continuity was included as an ‘add-on’ rather than being specifically explored *per se*. Moreover, the findings are further limited by the small number of studies that met the inclusion criteria, with many studies rejected on the basis that they did not explicitly identify continuity. This lack of systematic exploration in a remote care context is an important baseline finding and emphasises the need to continue to encourage those working in primary care to defend important values and practices such as continuity by stronger means than simply (as one sceptical editor once wrote) *‘invoking its warm, fuzzy heart, beating away in its black box, far from the close scrutiny of all but its adepts’.*^51^

### Comparison with existing literature

In 2010, Freeman and Hughes identified aspects of general practice that promoted high-quality continuity, and flagged some challenges.^7^ In parallel to the themes here, they highlighted the value that many patients place on continuity in primary care depending on their characteristics, circumstances, and reason for consulting. Those with serious or chronic conditions, older people, and those who are vulnerable or in poor health value it more,^52^ with others prioritising quick and convenient access.^53^

Freeman and Hughes specifically acknowledged the importance of access processes, practice systems (such as usual doctor lists), and task distribution (for example, long-term condition management by specialist nurses, and urgent issues by acute care teams) as crucial factors in facilitating or hindering high-quality continuity. Interestingly, they noted that, of the six case study practices they explored, none included specific statements about continuity in their literature or websites, consistent with the tacit assumption in some studies here about its importance.^7^

However, when the primary studies in the 2010 review were conducted, only 12% of UK GP consultations were carried out by phone,^54^ and email was sporadic.^55^ While earlier studies have highlighted how remote approaches can be used to promote continuity, for example, for follow-up or as a convenient alternative to seeing the usual GP in person,^56^ the rapid, widespread use of remote consultations brought about by COVID-19 did not allow for strategic implementation, nor has there been systematic assessment of its impact. Moreover, the expansion of remote modalities has been part of a wider pandemic-driven shift towards technology-mediated care, using websites, national telephone advice lines, and virtual wards,^57^ which is likely to have further complicated the picture.

### Implications for research and practice

The COVID-19 pandemic has offered an opportunity to explore widespread use of alternative consultation approaches.^58^ However, it has also forced a consideration of what is truly important in general practice and health care more widely.

In adapting to the ‘new normal’ it will be important to assess the impacts of ongoing remote approaches while exploring how they may be appropriately deployed. Given the benefits of continuity, optimising its delivery in those contexts where it is most important and valued may be considered an ‘internal good’ (the practice of continuity itself may result in positive value(s) in and of itself) in the practice of medicine.^59^ Where continuity is desirable, its absence may contribute to moral injury (a sense of harm arising from a challenge to an individual’s core values); burnout, and reduced retention among professionals; direct harm and/or structural violence (where individuals may perceive harm because of the way society or institutions are structured) towards patients; and overall system failure.

If continuity is compromised for those with complex and chronic conditions or for individuals experiencing a change in health status in order, for example, to prioritise convenience, ease, and rapidity of access for the digitally enabled, GPs risk becoming transactional specialists in minor illness and gatekeepers for siloed complaints (where inter-related multimorbidity is simplified into single health issues), rather than expert generalists with a holistic oversight of an individual’s health, wellbeing, and narrative. This could undermine the health-promoting potential of person-centred, longitudinal care,^60^ increasing demand on overstretched healthcare systems, and eroding the social and structural support primary care offers the wider community.

Currently, there is a malignant normality associated with these changes in practice.^61^ (This reflects a negative cycle where implementation of processes resulting in the loss of continuity and the associated impacts become accepted as normal. Ultimately, unless challenged, this may result in a pervasive erosion of values and practices causing further harms to individuals and society.) It is essential the counterfactuals (such as, for example, explicitly highlighting that promotion of rapid access in a resource-constrained system may occur at the expense of relational continuity and the associated negative consequences, and it is essential to explore whether this is the most desirable outcome from our healthcare system) are explicitly stated, systematically explored, and implications discussed — not just among academics, but also among practitioners and patients too. Moreover, oversimplification must be avoided. Instead, real-world explanatory frameworks that acknowledge the contextual complexity around adopting new practices should be considered.

One such approach, the planning and evaluating remote consultation services framework,^62^ combined with in-depth qualitative and ethnographic methodologies, is currently being used in the RbD2 study (and an associated PhD project) to explore the contextual, technological, wider system factors, and underpinning principles that enable and support or hinder the clinical relationship that sits at the heart of any healthcare encounter. This, and similar approaches, may help identify how the interacting influences on remote consultations play out in practice using methodologies that can capture complexity. This should allow for their optimal deployment that avoids devaluing the bedrock principles of general practice.
